# Shape Complementarity Optimization of Antibody–Antigen Interfaces: The Application to SARS-CoV-2 Spike Protein

**DOI:** 10.3389/fmolb.2022.874296

**Published:** 2022-05-20

**Authors:** Alfredo De Lauro, Lorenzo Di Rienzo, Mattia Miotto, Pier Paolo Olimpieri, Edoardo Milanetti, Giancarlo Ruocco

**Affiliations:** ^1^ Department of Sciences, Roma Tre University, Rome, Italy; ^2^ Center for Life Nano & Neuro-Science, Istituto Italiano di Tecnologia, Rome, Italy; ^3^ Department of Physics, Sapienza University, Rome, Italy

**Keywords:** antibobies, shape complementarity, molecular interaction, SARS-CoV-2, computational modeling

## Abstract

Many factors influence biomolecule binding, and its assessment constitutes an elusive challenge in computational structural biology. In this aspect, the evaluation of shape complementarity at molecular interfaces is one of the main factors to be considered. We focus on the particular case of antibody–antigen complexes to quantify the complementarities occurring at molecular interfaces. We relied on a method we recently developed, which employs the 2D Zernike descriptors, to characterize the investigated regions with an ordered set of numbers summarizing the local shape properties. Collecting a structural dataset of antibody–antigen complexes, we applied this method and we statistically distinguished, in terms of shape complementarity, pairs of the interacting regions from the non-interacting ones. Thus, we set up a novel computational strategy based on *in silico* mutagenesis of antibody-binding site residues. We developed a Monte Carlo procedure to increase the shape complementarity between the antibody paratope and a given epitope on a target protein surface. We applied our protocol against several molecular targets in SARS-CoV-2 spike protein, known to be indispensable for viral cell invasion. We, therefore, optimized the shape of template antibodies for the interaction with such regions. As the last step of our procedure, we performed an independent molecular docking validation of the results of our Monte Carlo simulations.

## 1 Introduction

Cellular functioning is widely dependent on processes occurring when biological molecules recognize each other and bind (Jones and Thornton, 1996; Gromiha et al., 2017). In particular, the non-covalent protein–protein pairing proved to be essential in several biochemical pathways, ranging from biocatalysis to organism immunity or cell regulatory network construction (Gavin et al., 2002; Han et al., 2004). Not surprisingly, in the last few decades, a very high amount of effort has been devoted to developing computational tools for the structural characterization of protein–protein complexes. The aim of these methods are various, varying from binding site identification (Gainza et al., 2020; Milanetti et al., 2021a) to binding affinity prediction (Vangone and Bonvin, 2015; Siebenmorgen and Zacharias, 2020) or protein–protein docking guide (Vakser, 2014; Kozakov et al., 2017; Geng et al., 2020; Yan et al., 2020).

In this scenario, the shape complementarity at the molecular interface is one of the most basic tasks to take into account (Katchalski-Katzir et al., 1992; Lawrence and Colman, 1993; and Jones and Thornton, 1996). Indeed, the evaluation of shape complementarity is essential for docking, both in terms of searching and evaluating the binding poses (Chen and Weng, 2003; Nicola and Vakser, 2007; Kuroda and Gray, 2016; Gromiha et al., 2017; and Yan and Huang, 2019), and represents one of the factors to take into account for binding site recognition (Gainza et al., 2020; Milanetti et al., 2021a) or to assess the binding affinity (Erijman et al., 2014).

Among the wide variety of methods developed in the last few years to describe the geometrical properties of a molecular region and to evaluate the complementarity with a putative binding partner region, using the Zernike polynomials is an effective and promising strategy (Venkatraman et al., 2009a; Di Rienzo et al., 2017; Daberdaku and Ferrari, 2019; and Di Rienzo et al., 2021a). Indeed, once extracted, the molecular surface region and its geometrical properties are summarized through a set of numerical descriptors, namely, the Zernike descriptors. The accuracy of the description is increased by enlarging the number of descriptors considered (Zernike and Stratton, 1934; Canterakis, 1999; and Novotni and Klein, 2004).

The main advantage of the Zernike formalism is that the molecular surface representation is invariant under protein rotation, constituting an absolute morphological characterization of the examined protein region. Therefore, the complementarity between two molecular regions is computed by comparing their Zernike descriptors, without the need for any preliminary superposition step (Daberdaku and Ferrari, 2018; Di Rienzo et al., 2020a).

In the last decade, the Zernike approach, in its 3D version, has been widely applied for the analysis of biomolecules (Venkatraman et al., 2009a; Venkatraman et al., 2009b; Kihara et al., 2011; Di Rienzo et al., 2017; Daberdaku and Ferrari, 2018; Daberdaku and Ferrari, 2019; Han et al., 2019; Di Rienzo et al., 2020a; Alba et al., 2020; and Di Rienzo et al., 2020b), proving its efficacy in characterizing both global and local protein properties.

We recently developed a computational protocol that allows us to employ the 2D Zernike formalism to assess the shape complementarity observed in protein–protein interfaces (Milanetti et al., 2021a). The utilization of the 2D formalism allows to sensibly decrease the computational time needed to compute the shape descriptors without a significant loss in description accuracy (Di Rienzo et al., 2021b).

In this work, we focused on antibody–antigen interactions, since these complexes represent a critical case of molecular recognition where the interface shape complementarity level is similar to the typical protein–protein interfaces (Li et al., 2003; Kuroda and Gray, 2016).

Moreover, antibodies have been the object of extensive biomedical studies since their modular architecture facilitates the engineering of novel binding sites (Gotwals et al., 2017; Saeed et al., 2017; and Singh et al., 2018). Indeed, the recognition of virtually any foreign antigen is due to high sequence variability in the antigen-binding site, while the overall architecture is largely conserved (Chothia and Lesk, 1987; Chothia et al., 1989; and Tramontano et al., 1990). The antigen-binding site is structurally composed of three loops of both the heavy and light chains, forming the *Complementary Determining Regions* (CDRs). Notwithstanding the variability of the CDR sequences, these loops (at least five out of six) can acquire only a limited number of structural conformations, called *canonical structures*. Moreover, studying the growing number of experimentally determined antibody structures, the relationship between the presence of given residues in certain sequence positions and the canonical structure adopted by the antibody has been demonstrated (Tramontano et al., 1990; Chothia et al., 1992; Foote and Winter, 1992; Decanniere et al., 2000; Chailyan et al., 2011; and North et al., 2011).

In this framework, thanks to the public availability of an increasing number of experimental antibody structures (Dunbar et al., 2013), several effective computational approaches—for predicting the structure of antibodies from their sequences—have been produced, often based on machine learning approaches (Dunbar et al., 2016; Lepore et al., 2017; Weitzner et al., 2017; and Abanades et al., 2022). Moreover, obtaining structural information about antibody–antigen complexes has been the object of extensive studies and it is still elusive. Many computational protocols focus on the prediction of the residues involved in partner interaction, both on the antibody and antigen side of the interface (Olimpieri et al., 2013; Potocnakova et al., 2016; Liberis et al., 2018).

All these kinds of computational tools can be used for antibody design, that is, the development of a novel molecule able to bind a given antigen (Norman et al., 2020). In particular, *ab initio* protocols are able to design paratopes integrating antibody structure prediction, molecular docking, and binding energy assessment (Pantazes and Maranas, 2010; Li et al., 2014; Lapidoth et al., 2015; and Adolf-Bryfogle et al., 2018).

Here, we collected a structural dataset of antibody–protein complexes solved in x-ray crystallography. In this work, we apply, for the first time, our recently developed method based on 2D Zernike descriptors to study the antibody–antigen interfaces. Concerning this specific kind of interaction, we demonstrate that such a fast and compact description can recognize with satisfying success the specific interaction from non-specific ones. Indeed, paratopes show a shape complementarity statistically higher toward their corresponding epitopes than toward epitopes belonging to unrelated antigens.

Based on these results, we propose here, for the first time, a new computational protocol employing 2D Zernike descriptors that, for a given target protein region, optimizes the shape complementarity of an antibody toward that region. Indeed, once a target region belonging to a protein antigen is identified and characterized with its Zernike descriptors, we compared such a region with the paratope of the antibodies in our dataset. Selecting as the starting template the antibody that has the most complementary patch, we perform a Monte Carlo (MC) simulation for the optimization of the paratope’s structural conformation. Through extensive computational mutagenesis, substituting in each step an interacting antibody residue with a different random one, we accept or reject each mutation according to the gain in shape complementarity, as evaluated by the Zernike method (Di Rienzo et al., 2020b; Di Rienzo et al., 2021b). In this work, the combined application of both the 2D Zernike formalism and a Monte Carlo simulation allows a computationally fast and effective exploration of the space of the possible mutants.

In the current pandemic situation, the interactions between SARS-CoV-2 spike protein and human cellular receptors have been extensively studied through the 2D Zernike polynomials formalism (Milanetti et al., 2021b; Bò et al., 2021; Miotto et al., 2021; and Miotto et al., 2022). Therefore, despite the generality of such an approach, we selected as a target for the optimization protocol some surface regions of the SARS-CoV-2 spike protein. We discuss here the results we got. Indeed, elucidating the interaction mechanism between antibodies and viral proteins represents a fundamental element for developing new therapies.

## 2 Results and Discussion

### 2.1 Description of the Antibody–Antigen Interface Through Zernike Descriptors

In the present section, we discuss the results we obtained applying our recently developed computational protocol (Milanetti et al., 2021a) on a structural dataset composed of 229 antibody–antigen complexes (see Methods).

**FIGURE 1:**
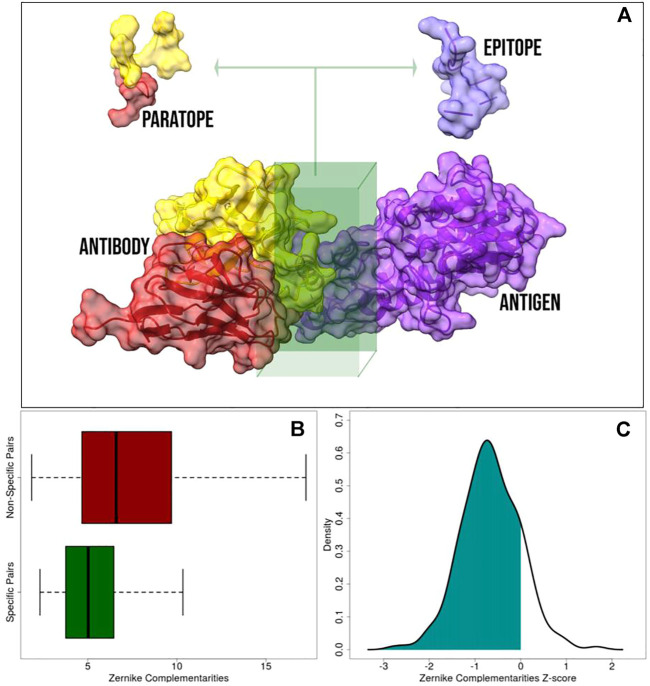
Application of the 2D Zernike polynomials approach to antibody–antigen complexes. **(A)** Representation of a molecular antibody–antigen complex: the antibody heavy chain, antibody light chain, and the antigen are in red, yellow, and purple, respectively. The interacting regions, defined as the portion of the molecular surfaces belonging to residues closer than 4 $\overset{{}^{\circ}}{A}$ to any atoms of the molecular partner, are extracted from the whole surface. **(B)** Boxplot comparing the *specific complementarity*, i.e., the complementarity between regions actually found in interaction (green), and the *non-specific complementarity*, i.e., the complementarity observed between paratopes and epitopes of different complexes (red). It is worth remarking that when the numerical value is low, the complementarity is high. **(C)** Z-score distribution of the specific complementarity. When the Z-score is lower than 0, the specific interaction is characterized by a complementarity higher than the mean of the non-specific interaction.

In particular, we firstly identified for each complex the paratope (epitope) as the set of residues with at least one atom closer than 4 $\overset{{}^{\circ}}{A}$ to an antigen (antibody) atom. Therefore, after separately computing the molecular surface (Richards, 1977) for both the proteins in interaction, we extracted the portions of the molecular surfaces relating to the binding site residues to properly characterize the shape of the interacting regions of antibodies and antigens (see Figure 1A).

Once we identified the interacting regions, we characterized them through the 2D Zernike polynomials, summarizing their geometrical properties in an ordered set of numerical descriptors (see Materials and Methods). By definition, two perfectly fitting surfaces are characterized by the same shape, meaning that, in principle, the difference between their Zernike descriptors is zero. Therefore, the shape complementarity between two molecular regions is compactly evaluated by such formalism. The lower is the distance between the Zernike descriptors and the higher is the shape complementarity between the corresponding protein regions (Milanetti et al., 2021a; Di Rienzo et al., 2021b).

We described all the paratopes and epitopes in the dataset with the Zernike formalism. In summary, we deal with 229 (the number of structures in our dataset) sets of 121 (the number of invariant descriptors when the order of expansion is set to 20) numerical descriptors for the paratopes and 229 for the epitopes. We thus defined the *specific complementarities* as the Euclidean distance between the descriptors of the 229 pairs of interacting (extracted from the same structure) paratope and epitope. Diversely, *Non-Specific complementarity* is the Euclidean distance between all the pairs of unrelated paratopes and epitopes (i.e. a paratope extracted from one structure and an epitope extracted from another one). In the end, we deal with 229 specific complementarities (one for each complex) and a high number (25,000) of non-specific complementarities (all the possible paratope–epitope pairs given a dataset of 229 items). In other words, in dealing with *N* antibody–antigen complexes, the specific complementarity, *C*_*s*_, is defined as

$$D\left(p_{i},e_{j}\right){|}_{i=j}=\sqrt{\overset{121}{\underset{k=1}{\sum }}{\left(p_{i}^{k}-e_{j}^{k}\right)}^{2}}{|}_{i=j},$$ (1)

where *D* is the Euclidean distance between the vectors of the paratope (*p*_*i*_) and epitope (*e*_*i*_) Zernike descriptors. Since we expanded all the paratopes and epitopes to the order 20, we dealt with 121 descriptors for each binding region. On the other hand, the non-specific complementarities, *C*_*ns*_, can be computed as

$$D\left(p_{i},e_{j}\right){|}_{i\neq j}=\sqrt{\overset{121}{\underset{k=1}{\sum }}{\left(p_{i}^{k}-e_{j}^{k}\right)}^{2}}{|}_{i\neq j}.$$ (2)

In Figure 1B, we reported a boxplot highlighting the differences between *C*_*s*_ and *C*_*ns*_ distributions. As expected, the distribution of *C*_*s*_ is statistically lower than the distribution of *C*_*ns*_ (Kolmogorov–Smirnov test *p* value <2.2e−16 ), testifying the sensitivity of Zernike in recognizing regions actually in contact from the non-interacting ones. In the second step, we normalized the Zernike complementarities with the Z-score. In particular, for each paratope we have 1 *C*_*s*_ and 228 *C*_*ns*_ complementarity values, each of which is related to a specific patch pair. Normalizing over this set of numbers and looking at the Z-score regarding the specific one, we assessed the propensity that characterizes each antibody toward its specific antigen. In Figure 1C, we report the distribution of such specific Z-scores. As evident, they are mostly negatives (86% of the cases have a Z-score lower than 0, and 28% have a Z-score lower than -1), providing evidence that specific interactions are characterized by lower distances (higher complementarity) than non-specific ones.

Taken together, these results confirm the ability of our method to correctly capture the main determinant of molecular shape complementarity.

### 2.2 Zernike-Based Monte Carlo Simulation for Molecular Interface Optimization

The Zernike formalism enjoys several advantageous features in representing the molecular surface: mainly, the invariance under rotation that makes such descriptors an “absolute” characterization of local protein morphology and the low computational cost of its calculation. Indeed, in this section, we present our algorithm that exploiting these advantages aims to optimize the shape complementarity of an antibody toward a given molecular target region. A similar procedure has already been presented and tested in our previous work (Di Rienzo et al., 2020b), and here for the first time, it is applied to antibody–antigen interaction systems.

**FIGURE 2:**
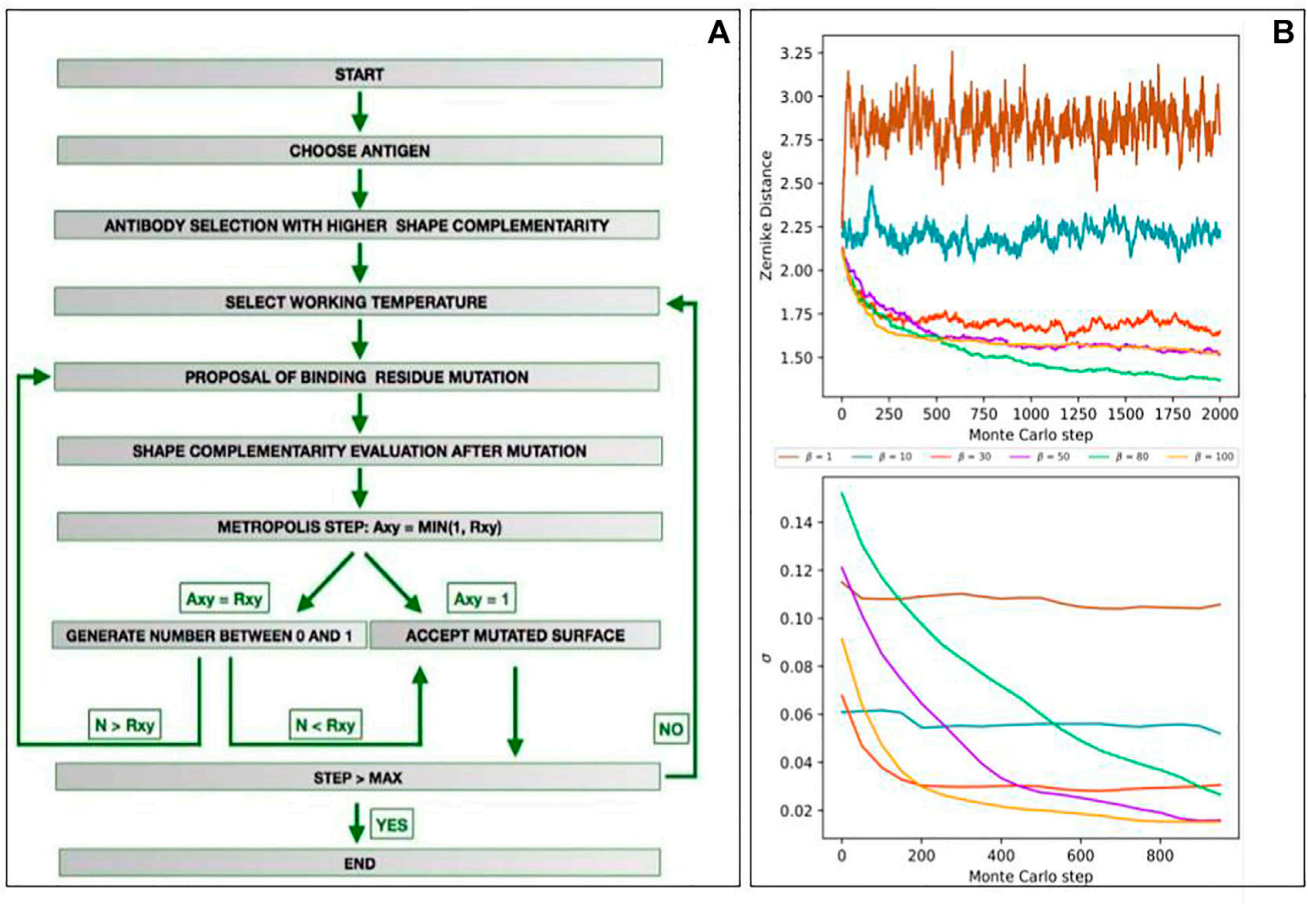
Development of a Monte Carlo simulation for shape complementarity optimization against a target region. **(A)** Flowchart depicting the main steps of the computational protocol we developed. **(B)** Results of the mutagenesis Monte Carlo procedures performed at fixed *β* (*β* = 1, 10, 30, 50, 80, and 100). The top panels represent the energy (i.e., the shape complementarity) as a function of the Monte Carlo steps. The low panels show the standard deviation of energy of the remaining part of the Monte Carlo simulations as a function of the Monte Carlo steps (i.e., *σ*(*E*) for n = 1,000 means standard deviations of the energy obtained in the steps 1,001–2,000).

Figure 2A illustrates the main steps of the algorithm. We defined the target region as the portion of the antigen surface toward which an antibody will be optimized. It is thus necessary to identify the antibody chosen as a starting point of the algorithm. Summarized with the Zernike descriptors of the target region we compute the shape complementarity with all the paratopes of our dataset; here, the template, i.e., the antibody selected as the starting point for the mutagenesis process, can be chosen among the paratopes characterized by a high initial complementarity.

After establishing the template, we perform a Monte Carlo simulation employing computational mutagenesis on the paratope residues. In each step, we randomly selected a residue mutating it into another one of the 19 possible ones. The mutation generates a different paratope, characterized by a different shape of its molecular surface. Consequently, re-computing the Zernike descriptors, we can evaluate the effect of the mutation on the complementarity with the target region; indeed we can define the *complementarity balance* as

$$\Delta C=C_{mut}-C_{wt}=D\left(p_{mut},e_{tar}\right)-D\left(p_{orig},e_{tar}\right),$$ (3)

where *p*_*orig*_ and *p*_*mut*_ are the Zernike descriptors of the original and the mutated paratopes, respectively, while the *e*_*tar*_ represent the Zernike descriptors of the target epitope and *D* represent the distance between 2 sets of descriptors. Since, as said, a high complementarity is reached when *D* is low, Δ*C* < 0 means a higher complementary surface, and Δ*C* > 0 is obtained when the mutation is deleterious since it causes a worsening of the shape complementarity.

The number of combinations of possible mutations in an interacting region, composed usually of tens of residues, is huge. Therefore, to effectively sample the space of the possible mutants, we perform a Monte Carlo Metropolis simulation, iterating the procedure described previously, where the mutation in each step is accepted according to the following rules:

$$P=\left\{\begin{array}{c} 1\;\;\;\;\;\;\;\;\;if\;\Delta C<0\;\\ e^{-\beta \Delta C}\;\;if\;\;\;\Delta C\geq 0\; \end{array}\right.,$$ (4)

where $\beta ∼\frac{1}{T}$ is the temperature factor that determines the probability of acceptance of a step worsening complementarity. Note that this aspect is crucial to properly exploring a large number of different mutants: *β* is thus progressively increased during the Monte Carlo simulation, progressively confining the system to minimum energy in a simulated annealing process (Kirkpatrick et al., 1983).

To observe how many steps are necessary to reach the equilibrium for each *β*, we preliminary ran several fixed-temperature Monte Carlo simulations. We selected the epitope of an antigen structure in our dataset (PDB id: 1AR1) and, excluding its one, we choose as the starting template, the most complementary paratope in the structural dataset. We thus performed six different Monte Carlo simulations, each for a different *β*, where the acceptation probability in each mutagenesis step is given by Eq. 4. Performing 10 independent simulations of M = 2,000 steps for each *β*, the averaged results we obtained are summarized in Figure 2B. In the top panel, we reported the energy (i.e., the complementarity) as a function of the Monte Carlo steps. In the low panel, we reported the standard deviation of the energy of the remaining part of the Monte Carlo simulations as a function of the number of steps (i.e., *σ*(*E*) for m = 1,000 means standard deviations of the energy obtained in steps 1,001–2,000). As expected, for low values of *β* (i.e., high temperature) the system lives in a condition of indifferent equilibrium, where whatever mutation has an equal likelihood to be accepted, independently from its effect on complementarity. When, on the contrary, *β* is high (low temperature), the energy of the system rapidly decreases to a stationary local minimum. This trend is confirmed by looking at the stationary value of energies or, equivalently, noting that standard deviation tends progressively to zero. In the light of these results, in our protocol, we set N = 700 for each temperature. In this way, we preliminary allow the system to move away from the starting local conformation, thus freezing it in a new energy minimum, characterized by an increased shape complementarity with the target region.

### 2.3 A Case of Study: Application to the SARS-CoV-2 Spike Protein

The approach described here is general and can be applied to whatever protein. This notwithstanding that we applied it to the SARS-CoV-2 spike protein is a very relevant case of macromolecular interaction. Indeed, the severe acute respiratory syndrome Corona virus 2 infection is still a very serious danger for public health (Huang et al., 2020; Zhu et al., 2020).

**FIGURE 3:**
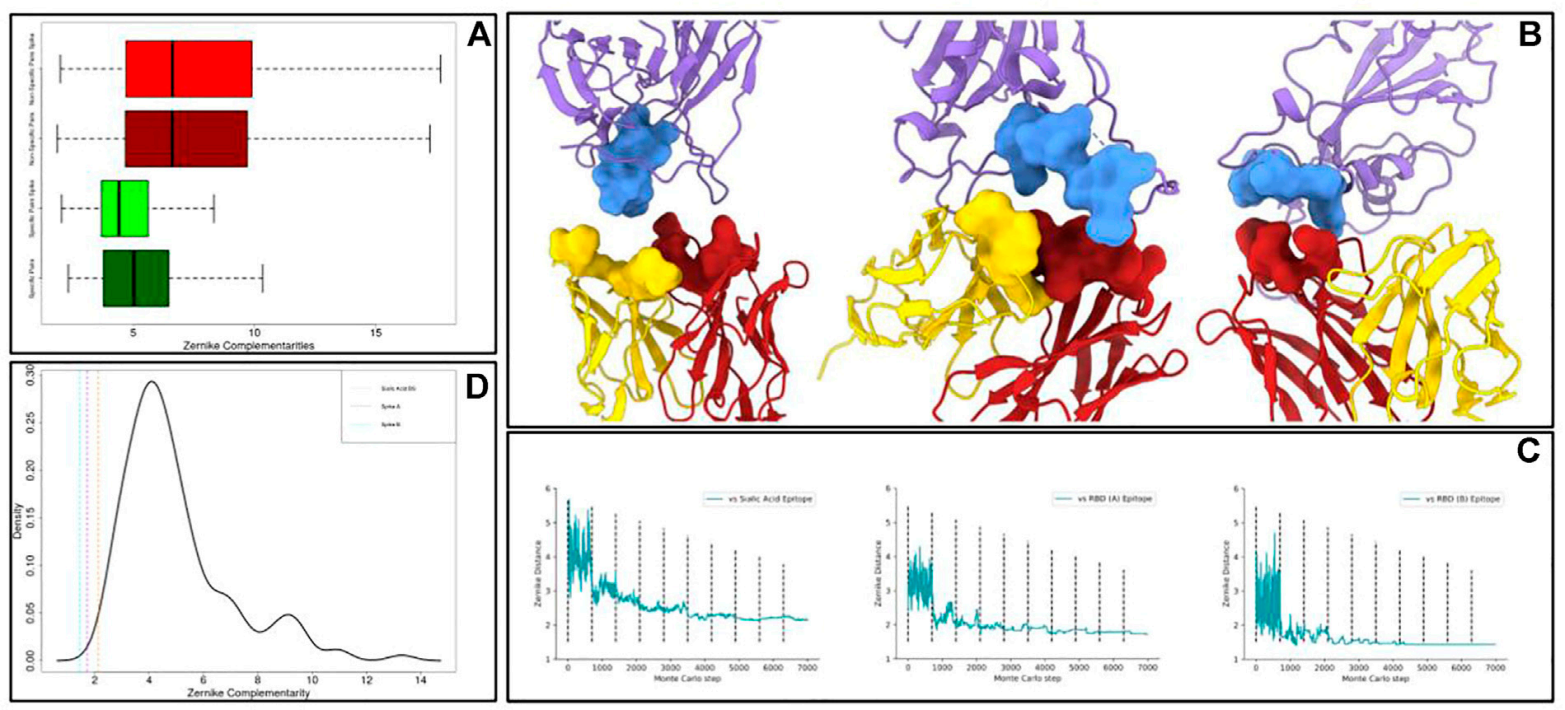
Application of the optimization protocol to the SARS-CoV-2 spike protein. **(A)** Boxplots comparing the *specific complementarity* and the *non-specific complementarity* in generic protein–antibody or in spike–antibody complexes. It is worth remarking that when the numerical value is low, the complementarity is high. **(B)** Molecular representation of the optimized antibodies binding epitopes on the spike protein. The antibody light and heavy chains are shown in yellow and red respectively, while the antigen is in purple. **(C)** Shape complementarity as a function of the Monte Carlo steps for all the antibodies we optimized. Dashed lines separate different temperature intervals of the simulations. **(D)** Probability density function of specific complementarities in the Spike dataset. The dashed lines represent the shape complementarity levels reached after the optimization protocols.

Many therapeutic strategies are devoted to the SARS-CoV-2 spike protein, protruding from the viral envelope and responsible for the cell entry mechanism (Walls et al., 2020; Wan et al., 2020; and Zhou et al., 2020). Thus, we obtained, using the dedicated section of Cov-AbDab (Raybould et al., 2021), a structural dataset of 145 spike–antibody complexes (we will call it the “Spike dataset”). We thus characterized the paratopes (antibody binding residues region) and the epitopes (various regions on the Spike) with the Zernike formalism. This allows us to compute, also for the Spike dataset, the *specific complementarities* and the *non-specific complementarities*, defined in Eqs 1, 2. The result of this analysis is shown in the following Figure 3A. These complementarities are reported as boxplots (light green for specific complementarities, light red for non-specific ones), even showing the boxplots regarding the general dataset (dark green and dark red, already shown in Figure 1B). Its results are evident that, also in the Spike dataset, specific interactions are characterized by a complementarity much higher than non-specific interactions (k.s. test *p*-value < 2.2 e-16). As expected, the non-specific interactions are represented by very similar distributions, since in both datasets they would represent an ensemble of non-interactions. Looking at these results, we noticed that the population of spike–antibody complexes showed the same behavior as the general population of the protein–antibody complexes.

In this framework, we selected on spike molecular surfaces, three different regions as targets for the optimization protocol. On one hand, we targeted two different molecular regions involved in the interaction between spike and angiotensin-converting enzyme 2 (ACE-2), the well-known cellular receptor responsible for viral cell invasion. Moreover, we also optimized an antibody toward a very exposed region in the N-terminal spike domain, responsible for contacting sialic acid molecules. Indeed, such an interaction can confer to the virus, as occurred for the Middle East respiratory syndrome coronavirus (MERS-CoV) (Li et al., 2017), an additional molecular mechanism for cell intrusion. The responsible spike region represents a promising therapeutic target (Baker et al., 2020; Milanetti et al., 2021b).

We selected the residues constituting such epitopes and we characterized their molecular surfaces through Zernike formalism. Thus, we calculated the complementarity between these regions and all the antibody binding sites in our original dataset. To begin the optimization from a favorite starting point, we selected as templates antibody-binding sites characterized by the highest complementarity with each identified target.

We applied the procedure described in the previous section obtaining optimized paratopes whose molecular images are shown in Figure 3B, where we reported both the optimized antibodies and antigen interacting surfaces. In Figure 3C, we reported, for each of the Monte Carlo simulations performed, the shape complementarity as a function of the steps of the simulation, where the dashed lines enclose ranges with different *β* values. Each simulation significantly optimizes shape complementarity, obtaining a Zernike distance decrease of 43% on average. Significantly, all the designed binding sites are characterized by a very high final shape complementarity, in terms of Zernike descriptors. Indeed, it is worth noting that the values obtained by all the three designed binding sites are lower than all the specific complementarities obtained in our structural dataset.

Moreover, in Figure 3D, we reported the probability density function of specific interactions in the Spike dataset. The vertical dashed lines represent the final complementarity values we get after the optimization procedures. It has to be noted that our protocol can effectively optimize the shape complementarity, obtaining final shape complementarity similar to the best cases observed in the Spike dataset.

**FIGURE 4:**
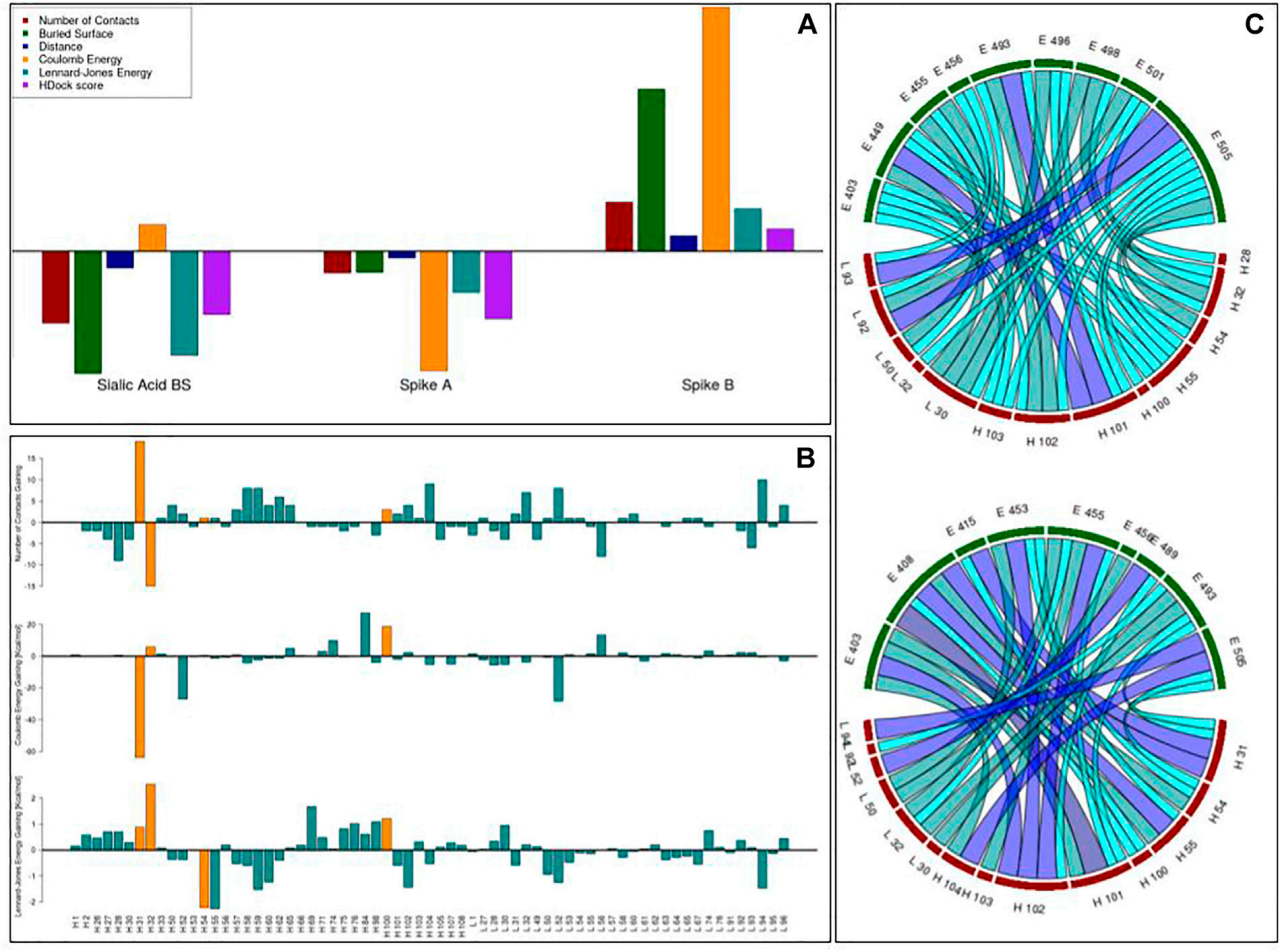
Results of the docking analysis. **(A)** Each bar represents the relative gaining (in terms of the number of residue–residue inter-molecular contacts, the surface buried in the complex, the mean inter-molecular distance of the closest atoms, the inter-molecular Coulomb energy, the inter-molecular Lennard–Jones energy, and the HDock binding score) between the 10 best docking poses obtained with the original and the optimized antibodies. A positive value means an increase in binding compatibility. **(B)** The gaining in terms of the number of inter-molecular contacts, Coulomb energy, and Lennard–Jones energy each residue registered before and after the optimization. **(C)** The network of residue–residue interactions at the interface when the original (upper figure) or the optimized (lower figure) antibody is docked to the spike B region. The color, from cyan to dark blue, and the width of the edges reflect the occurrences in the docking poses of a given contact.

The computational protocol we developed does not take into account several properties, known to be important in molecular recognition, such as electrostatics or hydrophobicity. In particular, our working hypothesis is that the shape complementarity plays a primary role as a perfect match between molecular surfaces due to an optimal structural rearrangement, which is probably caused by the compatibility of amino acid compositions of the interacting patches. However, the relationship between shape complementarity and chemical–physical properties is not always trivial, requiring a further test for the patches proposed as interacting, to also analyze the compatibility of a chemical nature. This means that a residue substitution can, in principle, worsen the chemical compatibility between molecules, even if the shape complementarity is enhanced. For this reason, as a further step of our optimization protocol, we performed a molecular docking analysis using HDOCK (Yan et al., 2020). More specifically, we docked the spike protein and the antibodies, both in the original and in the optimized versions, to study the effects our computational protocol has produced. We constrained docking to interact with the residues composing the spike target epitopes and the antibodies’ optimized regions. We summarized in Figure 4 the results we obtained.

Thus, we selected the 10 best docking poses regarding both the original and the optimized antibodies. To assess whether the optimization protocol has been effective, some estimators of binding compatibility have been calculated. In particular, the number of residue–residue inter-molecular contacts, the surface buried in the complex, the mean distance of the closest atoms between the two interfaces, the Coulomb inter-molecular energy, the Lennard–Jones inter-molecular energy, and the HDock binding score. For each of these observables, we computed the relative percentage of gaining after optimization so that the positive values indicate an increased binding tightness (See Materials and Methods). As shown in Figure 4A, even if in two applications we note a worsening, in one case, the optimization procedure has produced an antibody with better values of all such estimators, indicating the importance of including the molecular docking approach as a filter of selected patches based on geometric compatibility.

We focused therefore on this case and we analyzed how the introduced residue substitutions were responsible for this better compatibility. Analyzing the best docking poses, in Figure 4B, we reported the gaining in terms of the number of inter-molecular contacts and inter-molecular energy each residue registered before and after the optimization. Each residue is represented by a blue bar, while the residues mutated in the protocol are depicted in orange. As evident from the main effect regarding the residues “H 31” and “H 32” indeed, to increase the shape complementarity, the optimization protocol preferred to switch the exposition of these residues. It can be noted that the “H 31” residue, characterized by a high increase in the number of contacts, gains a very high amount of favorable (negative) Coulomb energy. Moreover, even if the number of inter-molecular contacts gained by H 54 is negligible, such a residue (and its neighborhood) acquired in the docking poses an increment of favorable Lennard–Jones energies.

Lastly, we assessed the difference in residue–residue inter-molecular interaction networks. In Figure 4C we reported the contacts between the main couples of residues, where a higher number of occurrences in the docking poses is testified by the thickness and the color of the edge. The spike residues are shown in green, and the antibody ones are in red. As further proof of the goodness of the proposed mutants, it can be noted that the interface of the optimized antibody (lower figure) is much more interconnected than the one of the original antibody (upper figure), indicating a possible effect on binding stability.

## 3 Conclusion

The binding affinity between biomolecules depends on a complex balance of several effects, including enthalpic and entropic contributions. Indeed, the substitution of even one residue at the interface could produce dramatic changes. Although many efforts were spent in this direction, predicting such effects has proven to be a difficult task and is still an open problem in computational biology.

In this scenario, the evaluation of shape complementarity between molecular regions is undoubtedly a central aspect. In this work, we focused on antibody–antigen interaction, a relevant case of molecular recognition. We applied our recently developed formalism based on the 2D Zernike polynomials to evaluate the shape complementarity with a quantitative approach. Once summarized the topological properties of interacting regions with a set of numerical descriptors, we demonstrated that such formalism assigns to pairs of interacting region complementarities statistically higher than the ones assigned to regions not in interaction.

We thus developed a Monte Carlo-based approach for the shape optimization of an antibody towards a molecular target region. We proposed a new strategy that, potentially, could modify an antibody in order to acquire a very high shape complementarity for a given epitope of any antigen–protein.

Because of the emergence of viral variants that can eventually escape antibodies maturated in vaccinated or recovered patients, the interactions between antibodies and SARS-CoV-2 spike protein are being extensively studied and still need further investigation.

For this reason, we selected three molecular regions on the spike protein as the target epitopes for our procedure. We, therefore, devised a set of antibodies characterized by a high shape complementarity toward their cognate epitopes.

However, even without considering therapeutically important elements such as immunogenicity and solubility, some other aspects have to be properly considered in our algorithm to increase the probability of identifying actually binding antibodies. Firstly, to produce more reliable mutant structures, the residue substitutions’ procedure has to account for the hypervariable loops canonical structure modeling. Moreover, we worked on antibody-bound structures: a structural conformational exploration can allow the antibodies to energetically rearrange their side chains, and to find the proper conformation able to bind the studied antigen. Finally, the binding compatibility does not depend only on shape complementarity, and thus the inclusion of terms accounting for the residues’ chemical characteristics will surely improve the method’s performance.

In conclusion, this procedure can represent a promising strategy for interface region molecular optimization, where the inclusion of the aspects discussed previously represents the necessary improvement steps. In the present work, we highlighted, with an independent molecular docking evaluation, the case when the optimization procedure has increased molecular complex compactness.

## 4 Materials and Methods

### 4.1 Dataset

We selected 229 protein-binding antibodies with a sequence identity lower than 90% and resolution <3.0 Å using the SabDab database (Dunbar et al., 2013). The Spike dataset, i.e., the structural dataset of the spike–antibody complexes was built using CoV-AbDab (Raybould et al., 2021). It results in 145 complex structures with a sequence identity lower than 90%, as calculated using CD-HIT (Huang et al., 2010).

The sequence of each antibody was renumbered according to the Chothia numbering scheme (Chothia and Lesk, 1987; Chothia et al., 1989) using an in-house python script.

The structure of the SARS-CoV-2 spike protein used for the identification of the ACE-2 interacting region has the PDB code 6vw1. When we investigated the N-terminal domain, we used the structure 7jji.

We identified on the spike protein two epitopes in the ACE-2 binding region: spike A and spike B. spike A epitope is constituted by the residues: “TYR 453, LEU 455, PHE 456, ALA 475, GLY 476, PHE 486, ASN 487, TYR 489, and GLN 493.” Spike B epitope is constituted by the residues: “TYR 449, GLY 496, GLN 498, THR 500, ASN 501, GLY 502, and TYR 505.” We identified another epitope in the N-terminal domain, in the region involved in sialic acid binding. That region is defined as the set of residues whose CA atoms are closer than $8\overset{{}^{\circ}}{A}$ to the TRP 258 CA (Milanetti et al., 2021b). Such an epitope is constituted by these residues: “LEU 244, HIS 245, ARG 246, SER 247, TYR 248, LEU 249, THR 250, PRO 251, GLY 252, ASP 253, SER 254, SER 255, SER 256, GLY 257, TRP 258, THR 259, and ALA 260”.

### 4.2 Surface Construction

Using as reference the experimental structures, computational mutagenesis has been performed using SCWRL4 (Krivov et al., 2009).

For each protein structure, solvent accessible surface is computed using DMS software with the standard option (Richards, 1977). The interacting surface is constituted by the surface points belonging to interacting residues, defined as the set of residues having at least one atom closer than 4 $\overset{{}^{\circ}}{A}$ to any atoms of the molecular partner.

### 4.3 Zernike Descriptors

Given a 2D function *f* (*r*, *ϕ*) in the unitary circle (region *r* < 1), it can be expanded in the Zernike polynomials basis. Therefore,

$$f\left(r,\phi \right)=\overset{\infty }{\underset{n=0}{\sum }}\overset{m=n}{\underset{m=0}{\sum }}c_{nm}Z_{nm},$$ (5)

where

$$\begin{array}{cc} c_{nm} & =\frac{\left(n+1\right)}{\pi }\left\langle Z_{nm}|f\right\rangle =\\ & =\frac{\left(n+1\right)}{\pi }\int_{0}^{1}drr\int_{0}^{2\pi }d\phi Z_{nm}^{*}\left(r,\phi \right)f\left(r,\phi \right) \end{array}$$ (6)

are the expansion coefficients (Zernike moments). The complex functions *Z*_*nm*_ (*r*, *ϕ*) are the Zernike polynomials, each composed of a radial and an angular part:

$$Z_{nm}=R_{nm}\left(r\right)e^{im\phi }.$$ (7)

The radial dependence, given *n* and *m*, can be written as follows:

$$R_{nm}\left(r\right)=\overset{\frac{n-m}{2}}{\underset{k=0}{\sum }}\frac{{\left(-1\right)}^{k}\left(n-k\right)!}{k!\left(\frac{n+m}{2}-k\right)!\left(\frac{n-m}{2}-k\right)!}r^{n-2k}.$$ (8)

For each couple of polynomials, the following rule holds:

$$\left\langle Z_{nm}|Z_{n^{\prime }m^{\prime }}\right\rangle =\frac{\pi }{\left(n+1\right)}\delta_{nn^{\prime }}\delta_{mm^{\prime }}.$$ (9)

Therefore, the set of polynomials forms a basis. Knowing the coefficients, {*c*_*nm*_} allows the reconstruction of the original function. The level of the detail can be modified by modulating the order of expansion, *N* = max (n).

The norm of each coefficient (*z*_*nm*_ = |*c*_*nm*_|) does not depend on the phase; therefore, it is invariant under rotations around the origin.

The shape complementarity between two regions can be evaluated by comparing their Zernike invariants. In particular, we measured the complementarity between regions *i* and *j* as the Euclidean distance between the invariant vectors, i.e.,

$$d_{ij}=\sqrt{\overset{M=121}{\underset{k=1}{\sum }}{\left(z_{i}^{k}-z_{j}^{k}\right)}^{2}}.$$ (10)

We adopted N = 20, therefore, dealing with 121 invariant descriptors for each patch.

### 4.4 Analysis of Docking Poses

In Table 1, we report the mutations proposed as a result of the three Monte Carlo simulations.

**TABLE 1:** The residue substitutions performed during the shape optimization procedure. The template structures for the sialic acid binding site, spike A, and spike B were 3bdy, 1yjd, and 1kb5, respectively. We adopted the Chotia numbering scheme.

Epitope	Chain ID	Res no.	Insert	Original res	Inserted res
Sialic acid BS	H	33	S	TYR	TRP
Sialic acid BS	H	52		TYR	TRP
Sialic acid BS	H	95		TRP	GLN
Sialic acid BS	H	100		PHE	ARG
Sialic acid BS	L	30	B	SER	ALA
Sialic acid BS	L	30	C	ILE	PHE
Sialic acid BS	L	30	D	SER	ILE
Sialic acid BS	L	31		TYR	ALA
Sialic acid BS	L	50		TRP	GLY
Sialic acid BS	L	91		HIS	SER
Sialic acid BS	L	92		TYR	GLY
Sialic acid BS	L	93		THR	HIS
Sialic acid BS	L	94		THR	VAL
Spike A	H	31		SER	LYS
Spike A	H	96		HIS	MET
Spike A	H	97		TYR	PHE
Spike A	H	98		GLY	LEU
Spike A	H	99		LEU	PRO
Spike A	H	100	S	ASP	HIS
Spike A	H	100	T	TRP	LYS
Spike A	L	30		TYR	ILE
Spike A	L	32		TRP	VAL
Spike A	L	91		GLY	ARG
Spike A	L	92		GLN	ARG
Spike B	H	31		GLY	GLU
Spike B	H	32		TYR	CYS
Spike B	H	53		TYR	PHE

We docked the three original and the three optimized antibody structures with spike using HDOCK (Yan et al., 2020), indicating as interacting residues the one written in the Dataset section.

We selected, for all the 6 docking simulations, the best 10 poses according to the Hdock binding score, an iterative knowledge-based scoring function. For each pose we get the following:

• The number of inter-molecular residue–residue contacts. Two residues are in contact if the minimum distance between their atoms is less than 4 $\overset{{}^{\circ}}{A}$ .
• The surface is buried in the complex. The surface buried is defined as the difference between the sum of the monomers’ area and the complex area. For this calculation, we use DMS software (Richards, 1977).
• The mean of the lowest 100 atom–atom inter-molecular distances.
• The sum of the Coulomb energy of the interactions occurring between antibody and spike atoms. We used the CHARMM27 force field (MacKerell et al., 2002).
• The sum of the Lennard–Jones energy of the interactions occurring between antibody and spike atoms. We used the CHARMM27 force field (MacKerell et al., 2002).
• The pose Hdock binding score.

The comparisons between the results regarding original and optimized antibodies are performed so as a positive value means an increase in binding compatibility after optimization. Therefore, the relative percentage of gaining is defined as follows:

• Number of contacts: $\frac{{<Cont>}_{opt}-{<Cont>}_{orig}}{{<Cont>}_{orig}}$

• Buried area: $\frac{{<Surf>}_{opt}-{<Surf>}_{orig}}{{<Surf>}_{orig}}$

• Distance: $\frac{{<Dist>}_{orig}-{<Dist>}_{opt}}{{<dist>}_{orig}}$

• Coulomb energy: $\frac{{<E_{c}>}_{orig}-{<E_{c}>}_{opt}}{{<E_{c}>}_{orig}}$

• Lennard–Jones energy: $\frac{{<E_{lj}>}_{orig}-{<E_{l}j>}_{opt}}{{<E_{l}j>}_{orig}}$

• HDock score: $\frac{{<Score>}_{orig}-{<Score_{c}>}_{opt}}{{<Score>}_{orig}}$

where the subscripts “orig” and “opt” refer to the poses obtained with antibodies before and after the optimization, respectively.

## Data Availability

All the protein structures are available on Protein Data Bank. The original contributions presented in the study are included in the article/Supplementary Material, further inquiries can be directed to the corresponding author.
